# Compound heterozygous *POMGNT1* mutations leading to muscular dystrophy-dystroglycanopathy type A3: a case report

**DOI:** 10.1186/s12887-019-1470-2

**Published:** 2019-04-08

**Authors:** Kondakova Olga Borisovna, Krasnenko Anna Yurievna, Tsukanov Kirill Yurievich, Klimchuk Olesya Igorevna, Korostin Dmitriy Olegovich, Davidova Anna Igorevna, Batysheva Tatyana Timofeevna, Zhurkova Natalia Vyacheslavovna, Surkova Ekaterina Ivanovna, Shatalov Peter Alekseevich, Ilinsky Valery Vladimirovich

**Affiliations:** 1 Scientific and Practical Centre of Pediatric psychoneurology of Moscow Healthcare Department, Michurinsky prospect, 74, 119602 Moscow, Russia; 2 Genotek Ltd, Nastavnicheskii pereulok 17/1, 105120 Moscow, Russia; 3 Pirogov Russian National Research Medical University, Ostrovitianova street 1, 117997 Moscow, Russia; 4 Institute of Gene Biology, Vavilova street 34/5, 119334 Moscow, Russia; 5 National Medical Research Centre for Children’s Health, Lomonosov prospect 2/1, 119296 Moscow, Russia; 6 Veltischev Research and Clinical Institute for Pediatrics of the Pirogov Russian National Research Medical University, Taldomskaya str 2, 125412 Moscow, Russia; 7 Institute of Biomedical Chemistry, Pogodinskaya street 10 building 8, 119121 Moscow, Russia; 8 Vavilov Institute of General Genetics, Gubkina street 3, 119333 Moscow, Russia

**Keywords:** Dystrophy-dystroglycanopathy, *POMGNT1*, MEB disease

## Abstract

**Background:**

Dystroglycanopathies, which are caused by reduced glycosylation of alpha-dystroglycan, are a heterogeneous group of neurodegenerative disorders characterized by variable brain and skeletal muscle involvement. Muscle-eye-brain disease (or muscular dystrophy-dystroglycanopathy type 3 A) is an autosomal recessive disorder characterized by congenital muscular dystrophy, ocular abnormalities, and lissencephaly.

**Case presentation:**

We report clinical and genetic characteristics of a 6-year-old boy affected by muscular dystrophy-dystroglycanopathy. He has severe a delay in psychomotor and speech development, muscle hypotony, congenital myopia, partial atrophy of the optic nerve disc, increased level of creatine kinase, primary-muscle lesion, polymicrogyria, ventriculomegaly, hypoplasia of the corpus callosum, cysts of the cerebellum. Exome sequencing revealed compound heterozygous mutations in *POMGNT1* gene (transcript NM_001243766.1): c.1539 + 1G > A and c.385C > T.

**Conclusions:**

The present case report shows diagnostic algorithm step by step and helps better understand the clinical and genetic features of congenital muscular dystrophy.

## Background

Congenital muscular dystrophy (CMD) is a clinically and genetically heterogeneous group of inherited muscle disorders characterized by hypotonia, delayed motor development and progressive muscle weakness. Muscle biopsies in patients with CMDs show considerable variability but always exhibit a characteristic pattern of dystrophic lesions. Disease typically manifests at birth or during the first year of life. The incidence of CMD in populations is not sufficiently known, there are small number of studies reporting incidence of CMD in several countries and regions: western Sweden (6.3 × 10^5^), Northern England (0.76 × 10^5^), north of Italy (4.7 × 10^5^) [1–3].

Progress in the field of molecular genetics allowed to determine genes whose mutated forms are responsible for CMDs. Identification of genetic cause and accurate diagnosis improve supportive therapy and family planning. But currently there is no specific treatment for this group of diseases. The most common CMDs are Ulrich congenital muscular dystrophy (UCMD, OMIM 254090) due to the pathology of collagen VI (mutations are found in three genes) [4], secondary dystroglycanopathies associated with a violation of α-dystroglycan (16 candidate genes) [5] and CMD caused by a primary merosin deficiency (merosin-deficient congenital muscular dystrophy, MDC1A) due to mutation of the LAMA2 gene [6].

Dystroglycanopathies are inherited in an autosomal recessive manner and characterized by brain and eye anomalies [7]. Clinical presentations can vary widely, from Walker–Warburg syndrome, a lethal perinatal form of congenital muscular dystrophy, to milder limb-girdle muscular dystrophy with manifestation in adulthood. To date, sixteen different genes are known as a cause of dystroglycanopathies: *POMT1, POMT2, POMGNT1, FKTN, FKRP, LARGE, DPM2, DPM3, DOLK, ISPD, GTDC2, TMEM5, B3GALNT2, B3GNT1, GMPPB, DPM1* [8]. There is only one population study in which Graziano with colleagues identified prevalence of dystroglycanopathies in Italy as 0.226 per 100,000 [9]. But this prevalence may vary in different populations, often due to founder mutations.

Dystroglycan is composed of two subunits (α and β) and is an essential component of the dystrophin-glycoprotein complex**:** linkage between the extracellular matrix and the membrane cytoskeleton in muscle fibers [10]. Destruction of the dystrophin-glycoprotein complex due to abnormalities in the structure of proteins or the violation of glycosylation leads to the development of muscular dystrophy. α-dystroglycan is also involved in neuronal migration, and its violation leads to anomalies in CNS [11].

Clinical findings of dystroglycanopathy include hypotonia starting in early infancy, marked mental and motor retardation, seizures, eye abnormalities (myopia, glaucoma, cataract, optic nerve atrophy, retinal dysplasia, microphthalmia). The following CNS abnormalities are observed: pachygyria, polymicrogyria, lissencephaly, hydrocephalus, ventriculomegaly, agenesis of the corpus callosum, hypoplasia of the brain stem, hypoplasia, dysplasia or cysts of cerebellum [7]. The most frequent dystroglycanopathies include muscle eye brain disease (MEB) [7], Walker-Warburg syndrome (WWS) [12] and Fukuyama congenital muscular dystrophy (FCMD) [13].

CMD diagnosis can be challenging, and in some cases patients with muscular dystrophy received therapy to treat perinatal CNS damage or other neurological diseases. Genetic testing allows to confirm CMD diagnosis and identify the disease at an early stage [14]. In this study, we analyzed clinical and genetic characteristics of a 6-year-old boy with muscular dystrophy-dystroglycanopathy. The symptoms his parents reported were fatigue during sports activities, stereotypes, lack of speech acquisition, gradual cognitive decline and unsteadiness.

## Case presentation

A 6-year-old boy was born of the second pregnancy and first childbirth to healthy nonconsanguineous parents. The first pregnancy ended in an early miscarriage. Family history is not burdened.

Pregnancy was with threatened spontaneous interruption. He was delivered by scheduled Cesarean section due to high myopia. His birth weight was 3650 g and height was 52 cm. Apgar scores were 8 and 9 at 1 and 5 min respectively. He was noted to have hypotonia at birth.

His motor milestones were delayed: held head by 7 months, rolled from back to side at 8 months, put into sitting position by 24 months, walked with support at 2,5 years and walked independently at 4 years. His speech development was delayed: at the age of 6 years he had no words.

At 8 month he was hospitalized to Scientific and Practical Centre of Pediatric psychoneurology. Brain computed tomography revealed signs of leukodystrophy and cortical atrophy of frontal, parietal and temporal areas. Radiological studies (neuroimaging) have never been performed until the age of 8 months.

At 13 months he was admitted to the National Medical Research Centre for Children’s Health and was monitored for the next 5 years.

At admission in 13 months brain MRI revealed signs of leukodystrophy, lissencephaly of left occipital lobe, polymicrogyria of both frontal lobes, secondary ventriculomegaly, brain atrophic changes with enlargement of sub-arachnoid spaces. Biochemical analysis showed creatine kinase (CK) level to be elevated to 2024 U/L (normal range 25–140 U/L), alanine aminotransferase (ALT) to 59 U/L (upper limit of normal 40 U/L), aspartate aminotransferase (AST) to 82 U/L (upper limit of normal 42 U/L) and lactate dehydrogenase (LDH) level to 318 U/L (upper limit of normal 225 U/L). On electromyographic examination, signs of primary muscle lesion have been identified.

**Fig. 1:**
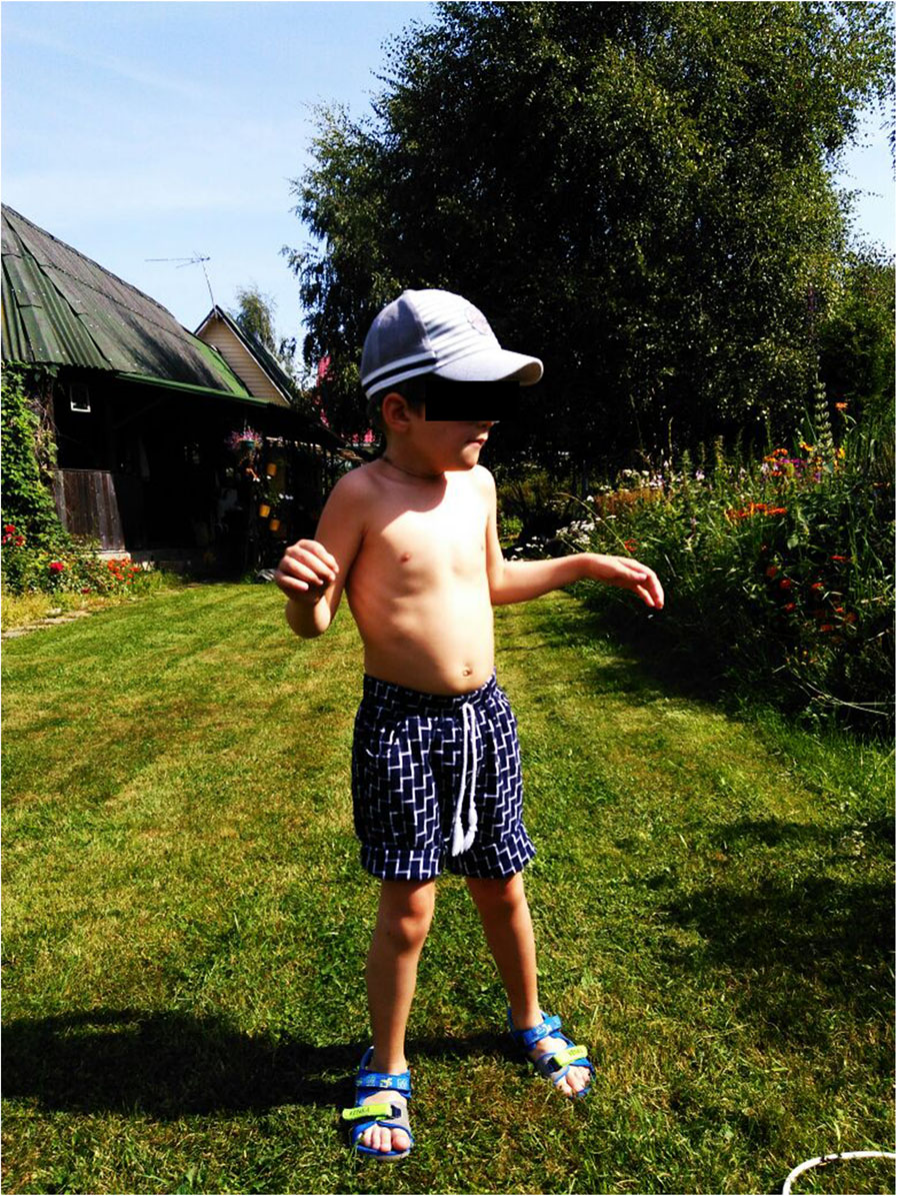
Photograph of the patient showing phenotype and stereotypic movements

At admission in 6 years weight was 21 kg, height was 113 cm, head circumference was 53 cm. Convergent strabismus, insufficiency of convergence and accommodation were noted. He had hypotonia, distal more than proximal. Trunk ataxia, walking on his toes with an atactic component, flat valgus foot were noted. Patient reacted favorably to examination. He can maintain short-term eye contact; speech is misunderstood. The face is hypomimic. In active speech there are long vocalized sounds, rarely - simple babbling syllables, no words. Behavioral features are manifested by periodic fading with subsequent rapidity of breathing and stereotyped swings of hands (Fig. 1).

Orthopedic examination revealed thoracic hypokyphosis, lumbar hyperlordosis, double-sided coxa valga, pathological antetorsion on the right, equinox flat valgus feet. Limb movements were not restricted except for restriction of back flexion in the ankles.

Ophthalmological examination revealed congenital high myopia, partial atrophy of an optic nerve and retinal atrophy in both eyes.

**Fig. 2:**
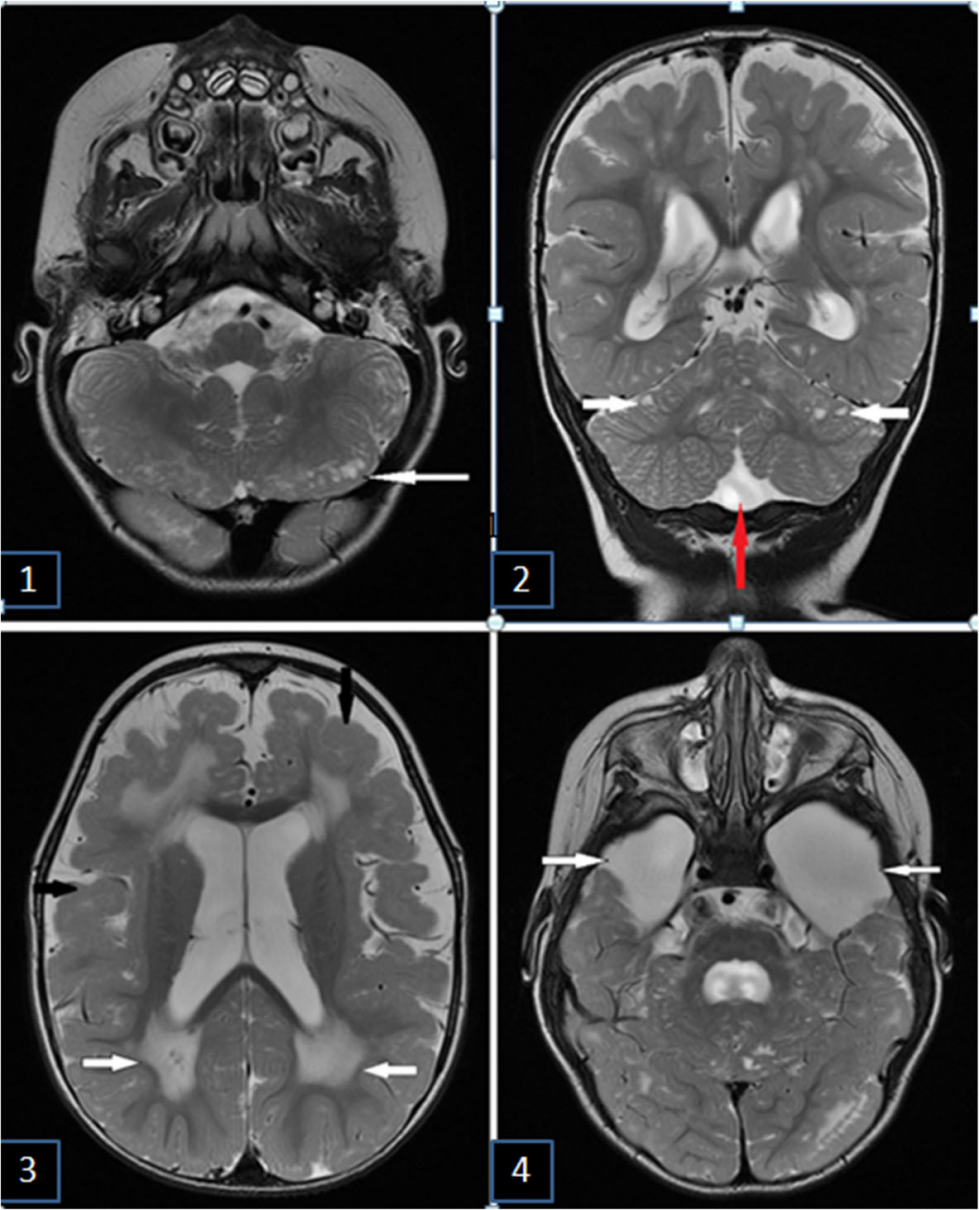
Brain MRI of the 6-year-old boy with dystrophy-dystroglycanopathy. **1** - Multiple subcortical cerebellar cysts (white arrow). Axial T2 weighted image. **2** - Hypoplasia of caudal parts of the cerebellum worm (red arrow) and subcortical cerebellar cysts (white arrows). Frontal T2 weighted image. **3** - Pachygyria-polymicrogyria of frontotemporal lobes (type “cobblestone pavement”) (black arrows) and demyelination of white matter (white arrows). Axial T2 weighted image. **4** - Hypoplasia of the temporal lobes (white arrows). Axial T2 weighted image

MRI revealed hypoplasia of temporal lobes and opercular areas from two sides, hypoplasia of caudal parts of the cerebellum worm with the expansion of external subarachnoid spaces of the cerebral hemispheres (more in the temporal areas), expansion of retrocerebellar cistern, symmetrical expansion of the ventricular system, pachygyria-polymicrogyria of cerebral hemispheres with preservation of the occipital lobe. Increased intensity of MR signal was observed in T2 and T2 FLAIR images. Decreased intensity of MR signal was observed in T1 image. In the cerebellum multiple small cysts, hypoplasia of the corpus callosum and pons with the extension of the anterior and covering cisterns are revealed (Fig. 2).

Biochemical analysis showed CK level to be elevated to 2253 U/L (normal range 25–194 U/L), ALT to 70,4 U/L (upper limit of normal 40 U/L), AST to 62 U/L (upper limit of normal 42 U/L) and LDH level to 503 U/L (upper limit of normal 314 U/L). Other blood and urine indicators were within normal limits. Patient level of CK was increased 10–15 times and for two years decreased from 3126 (3 years 4 months) to 1986 (at 5 years 2 months). The level of CK-MB was also increased - 61.1 (upper limit of normal 25 U/L).

On electroneuromyographic examination (ENMG), signs of primary muscle lesion have been identified. Motor unit potentials (MUP) were short in duration and low in amplitude. The number of polyphasic MUP has increased. Spontaneous activity in each test muscle was determined.

Electroencephalography (EEG) did not reveal epileptiform activity.

The electrocardiogram (ECG) showed a vertical position of the electrical heart axis, mild bradycardia and arrhythmia, nonspecific intraventricular block, early repolarization syndrome of the ventricles.

A standard karyotype was normal 46, XY. Tandem mass spectrometry did not reveal any changes in the plasma level of amino acids and acylcarnitines.

Genotek Ltd. did clinical exome sequencing. Genomic DNA from peripheral blood sample was extracted using QIAamp DNA Mini Kit (Qiagen) according to manufacturerʼs protocol. DNA libraries were prepared using NEBNext Ultra DNA Library Prep Kit for Illumina (New England Biolabs) with adapters for sequencing on Illumina platform according to manufacturer’s protocol. We used SureSelect XT2 (Agilent Technologies) for target enrichment. Enriched samples were sequenced on Illumina HiSeq 2500 using pair-end 100 base pairs reads. After sequencing, we trimmed 3′-nucleotides with read quality below 10 using Cutadapt [15]. Raw reads were aligned to reference genome hg19 (GRCh37.p13) using BWA MEM [16]. Deduplication of reads was done using SAMtools rmdup [17]. FastQS was used for data quality control [18]. We called short variants using GATK HaplotypeCaller [19] according to GATK Best Practices DNA-seq [20, 21]. The effect of each mutation was assessed using snpEff [22]. To assess pathogenicity and conservatism, the data was extracted from the dbNSFP [23], Clinvar [24, 25], OMIM database [26] and HGMD [27], as well as using the SIFT [28] and PolyPhen-2 [29, 30] utilities to predict pathogenicity of the mutation. Information on frequency of mutations was taken from 1000Genomes project [31, 32], ExAC [33, 34] and Genotek frequency data. Pathogenicity was predicted according to the Standards and Guidelines developed by ACMG (American College of Medical Genetics and Genomics), AMP (Association for Molecular Pathology) and CAP (College of American Pathologists) [35]. Copy number alterations were determined using CNVkit [36].

Sanger sequencing confirmed *POMGNT1* pathogenic variants identified by exome sequencing. For labeling amplicons with fluorescent labels, BigDye Terminator Cycle Sequencing Kit v3.1 (Thermo Fisher Scientific) was used. Sanger sequencing was performed on ABI PRISM 3500 Genetic Analyzer (Applied Biosystems) according to manufacturerʼs protocol.

After exome sequencing, patient was commenced on muscle relaxants and anticonvulsants. His condition is stable.

## Discussion and conclusions

**Fig. 3:**
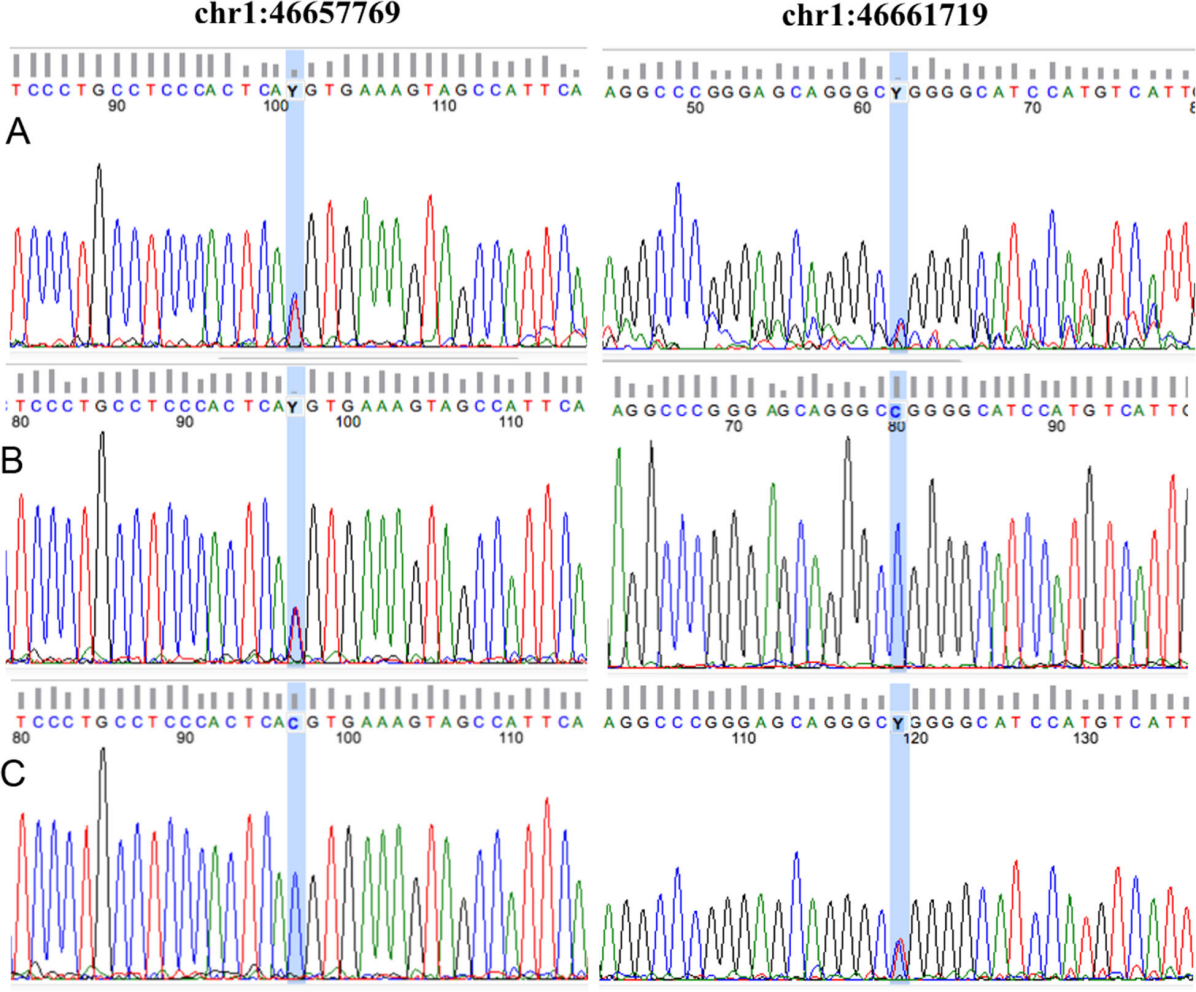
Sanger sequencing chromatograms of the proband (**a**) and his parents (**b** - father and **c** - mother). Sequencing showed the proband to be compound heterozygous for c.1539 + 1G > A and c.385C > T mutations of the *POMGNT1.* Mutation c.1539 + 1G > A present in the father and mutation c.385C > T present in the mother

In our patients, two heterozygous mutations c.1539 + 1G > A and c.385C > T of the *POMGNT1* gene were found by NGS analysis and confirmed by Sanger sequencing. First mutation c.1539 + 1G > A present in the father and second mutation c.385C > T present in the mother (Fig. 3). Thus, compound-heterozygous localization of these two mutations was confirmed. Mutations are described in the literature: c.1539 + 1G > A is a splice site pathogenic mutation in 17 intron [37–39], c.385C > T is likely pathogenic mutation in 5 exone.

Mutation c.1539 + 1G > A (chr1:46657769, genome build GRCh37) is a substitution in intron 17, which altered the conserved GT splicing donor sequence to AT. This mutation caused read-through of intronic sequences, resulting in the introduction of a premature termination codon. The mutation also caused skipping of the upstream exon 17, resulting in the deletion of 42 amino acids (p.Leu472_His513del) [37–39].

Mutation c.385C > T (chr1:46661719) is a missense mutation results in arginine substitution for tryptophan at position 129 (p.Arg129Trp).

Exome sequencing also revealed heterozygous deletion in the first intron of *FKTN* gene (IVS1-15delA, c.3515del A). According to the UCSC Genome Browser [40], this deletion is in the middle of the intron and does not affect the splicing sites. This mutation is not described in the literature and databases. There were no mutations in the exons of this gene. Mutations in *FKTN* are the main cause of the Fukuyama congenital muscular dystrophy. For all of these reasons, we defined this mutation as non-pathogenic.

Diagnostic algorithm for the detection of CMDs contains following steps: assessment of presence or absence of CNS, muscle and vision damage, as well as evaluation of phenotypic features and the plasma level of CK. In the case of CNS involvement, brain MRI is performed. The final step is a molecular study to confirm the diagnosis [41].

Ulrich’s disease is the most common form of CMD in Europe but does not cause CNS anomalies [42]. In our case, this type of CMD was excluded. The revealed by MRI complex of changes can be characteristic for congenital merosin-deficient myodystrophy, type 1A (OMIM 607855). But the specific damage to the organ of vision and absence of contractures of the joints make it possible to exclude this diagnosis. Elevated levels of CK in the patient’s blood and signs of CNS involvement suggested the presence of secondary dystroglycanopathy. MRI data confirm this diagnosis. Anomalies corresponding to secondary dystroglycanopathies and detected by MRI are disruption of the formation of brain gyri, combined with a flattening of the cortical pattern, an abnormality of the supratentorial white matter, cerebral cysts, and cerebellar hypoplasia [43].

Dystroglycanopathy is associated with an abnormal α-dystroglycan function. α-dystroglycan is one of the subunits of dystroglycan and central component responsible for the stability of the myocyte membrane [44]. α-dystroglycan is an extracellular membrane glycoprotein that binds to the extracellular matrix component - laminin. Β-dystroglycan binds to the protein of the cytoskeleton - dystrophin [45]. α-dystroglycan is composed of three domains: N-terminal, mucin-like and C-terminal domains. O-mannosylation of the mucin-like domain of α-dystroglycan plays an important role in the interaction with laminin. Defects in glycosylation of the α-subunit lead to a decrease in binding of the dystroglycan to extracellular matrix proteins, which leads to membrane fragility [46]. The most severe symptoms of dystroglycanopathy are observed with mutations in genes encoding proteins of the O-mannosylation process [47]. α-dystroglycan is also expressed in neurons and oligodendrocytes and participates in the formation of the brain during fetal development [48]. The role of α-dystroglycan in brain development is not fully understood, but it has been shown that it participates in the formation of basal membrane and migration of neurons [45].

Secondary dystroglycanopathies include MEB, WWS syndrome and syndrome FCMD. Genetic analysis was conducted to establish an accurate diagnosis among the variants described.

FCMD syndrome (OMIM 253800) is an autosomal recessive disease characterized by severe muscular dystrophy in combination with CNS lesions (brain polymicrogyria, lissencephaly, agenesis of corpus callosum, ventriculomegaly, cerebellar cysts and hypomyelinization) [49]. Clinical signs of the disease include the development of contractures of large and small joints, heart damage (congenital heart disease, dilated cardiomyopathy). Patients with FCMD syndrome also suffer from epileptic attacks (80%) and insomnia (30%). Also, they have visual problems, such as myopia, retinal dysplasia, atrophy of optic nerve, microphthalmia, cataract. The retardation of mental development in patients with FCMD syndrome is estimated from moderate to severe. Usually, such patients do not live up to 20 years. Mutations in *FKTN* are the main cause of the Fukuyama congenital muscular dystrophy. *FKTN* is located on chromosome 9q31 and encodes membrane protein that is believed to have a function similar to glycosyltransferase and may be involved in protein modification after the phosphorylation process [50]. The coding region of *FKTN* was analyzed by NGS. A nonpathogenic heterozygous deletion in the first intron of this gene was detected (IVS1-15delA, c.3515del A). Based on clinical signs of the patient (absence of contractures and congenital heart diseases) and absence of pathogenic mutations in the *FKTN* gene, the diagnosis of Fukuyama syndrome was excluded.

MEB disease (or muscular dystrophy-dystroglycanopathy type 3 A, OMIM 253280) is an autosomal recessive disease characterized by congenital muscular dystrophy, visual impairment and defects of brain development (lissencephaly). The average life expectancy of patients with MEB is 10 to 30 years [42]. The most common cause of MEB are mutations in the *POMGNT1*. All described *POMGNT1* mutations lead to complete loss of enzymatic activity of the POMGNT1 protein, responsible for the modification of α-dystroglycan after the phosphorylation process. Normally, it catalyzes the addition of the N-acetylglucosamine residue to O-linked mannose [37]. The coding region of *POMGNT1* was analyzed by NGS. Two mutations were found, in intron 17 (c.1539 + 1G > A) and in exon 5 (c.385C > T). Mutation c.1539 + 1G > A is found in splice site and is registered in the databases as pathogenic (https://www.ncbi.nlm.nih.gov/clinvar/variation/56582/). Mutation c.385C > T in the compound heterozygous state is registered with unknown effect (http://databases.lovd.nl/whole_genome/variants/0000067353#00014727).

Mutations in the *POMGNT1* can lead not only to MEB disease but also to the development of Walker-Warburg syndrome associated with congenital muscular dystrophy, violation of neuronal migration and various visual disorders [51, 52]. However, clinical manifestations (delay in psychomotor development, brain and eye damage) of Walker-Warburg syndrome are more severe. As a result, the average life expectancy of patients with Walker-Warburg syndrome is about three years [53, 54]. Based on clinical signs and age of our patient the correct diagnosis is muscular dystrophy-dystroglycanopathy type 3 A (MEB disease).

Thus, based on clinical features (severe delay in psychomotor and speech development, muscle hypotony, congenital myopia, partial atrophy of the optic nerve disc), increased level of CK, ENMG data (primary-muscle lesion), MRI data (polymicrogyria, ventriculomegaly, hypoplasia of the corpus callosum, cysts of the cerebellum) and results of molecular genetic analysis patient was diagnosed: сongenital muscular dystrophy-dystroglycanopathy, type 3A (MEB syndrome, with damage to the muscles, eyes and brain).

Identification of disease-causing mutations can provide a proper molecular diagnosis of dystroglycanopathies, which will help to consider more reliable therapeutic approaches and develop information about prognosis.

Currently, there is no specific treatment for dystroglycanopathies. Treatment of such patients focuses on decreasing the severity of symptoms. However, with the limited available therapeutic option for most of the patients, molecular diagnosis might enable geneticists and pediatricians to provide informative genetic counseling, perform prenatal diagnosis, and implement prevention measures for such patients. Therefore, genetic counseling should be recommended to all individuals with dystroglycanopathies and families for their next pregnancies.

In summary, two mutations of the *POMGNT1* gene were identified in a compound heterozygous boy with muscular dystrophy-dystroglycanopathy. We described detailed algorithm for correct differential diagnosis of dystroglycanopathies: MEB syndrome, WWS syndrome and FCMD. Our study may help to establish an appropriate genetic counseling and prenatal diagnosis for individuals at the high risk of dystroglycanopathy.

## Abbreviations

CK: Creatine kinase
CMD: Congenital Muscular Dystrophy
CNS: Central Nervous System
ECG: Electrocardiogram
EEG: Electroencephalography
ENMG: Electroneuromyographic examination
FCMD: Fukuyama congenital muscular dystrophy
MEB: Muscle Eye Brain disease
MRI: Magnetic Resonance Imaging
WWS: Walker-Warburg syndrome
